# Widespread Underestimation of Drought Impacts on River Ecosystems Due to Weak Experimental Designs

**DOI:** 10.1111/gcb.70993

**Published:** 2026-07-24

**Authors:** Eva Haristoy, Charlotte Evangelista, Mathieu Buoro, Stephanie M. Carlson, Arturo Elosegi, Cédric Tentelier, Albert Ruhí

**Affiliations:** ^1^ Université de Pau et Des Pays de L'Adour, INRAE, ECOBIOP Saint‐Pée‐sur‐Nivelle France; ^2^ Water and Biodiversity Division Norwegian Institute for Nature Research Torgarden Trondheim Norway; ^3^ Department of Environmental Science, Policy, and Management University of California, Berkeley Berkeley California USA; ^4^ Department of Plant Biology and Ecology, Faculty of Science and Technology University of the Basque Country (UPV/EHU) Leioa Spain

**Keywords:** BACI, biodiversity, drought, ecosystem functioning, experimental design, meta‐analysis, rivers

## Abstract

Hydrologic droughts are intensifying globally due to climate change and human water demand. Although a myriad of drought impacts on river ecosystems have been described, manipulative experiments on the topic have often yielded inconclusive or inconsistent results. Here, we investigated to what extent this inconsistency could be due to uncontrolled variation in methodological choices, or to variation in how different ecological endpoints respond to drought. To this end, we developed a meta‐analysis of 1284 effect sizes from 53 experiments, and estimated drought impacts on key variables of riverine biodiversity and ecosystem functioning. We evaluated study design (e.g., temporal vs. spatial focus, controls, treatment characteristics), focal ecosystem endpoint and variable type (i.e., quantity, structure, function), and level of biological organization studied. We found that methodological features strongly influenced the detectability and magnitude of the estimated drought effects, with Before‐After–Control‐Impact (BACI) designs reporting 2× stronger impacts than simpler designs. Effects differed among measures of quantity, structure, and function; and across ecosystem endpoints, with sensitivity to drought generally increasing with trophic position. Finally, for BACI designs, responses tended to be 1.5× weaker at higher levels of organization (ecosystem) relative to lower levels (population), suggesting an important role of compensatory dynamics and functional redundancy in absorbing drought impacts. Our findings confirm that drought degrades river ecosystem structure and functioning, but effect detectability and magnitude strongly depend on experimental choices and ecological focus. Although studies with replication over space and time (e.g., BACI) require more effort, we show that they are uniquely powerful in parsing out true drought effects from confounding effects of other time‐varying processes. We contend that embracing robust experimental designs that include spatio‐temporal controls and multiple levels of organization would vastly improve our ability to forecast ecological consequences of global change in river ecosystems.

## Graphical abstract

Experimental design strongly influences the detectability and magnitude of estimated drought impacts. Simpler designs underestimate impacts relative to the more complex, but relatively rare (< 35% of the studies), Before‐After–Control‐Impact designs.

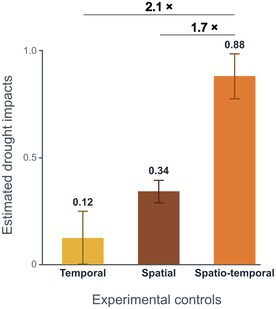

## 1 Introduction

River ecosystems play a disproportionately large role in global nutrient cycling and the maintenance of biodiversity (Heathwaite 2010; Abell et al. 2008), but these services are threatened by climatic droughts and human‐induced water scarcity (Vörösmarty et al. 2010; Sabater et al. 2018; Battin et al. 2023). Recent observations, supported by climate model hindcasts and forecasts, indicate that drought episodes are becoming longer and more frequent with climate change (Seneviratne et al. 2021), even in free‐flowing streams (Carlson et al. 2024; Gudmundsson et al. 2025). Drought episodes impact river ecosystems in important ways, such as by contracting wetted habitat and by warming up and deoxygenating stream water (van Vliet et al. 2013; Sprague 2005; Zhi et al. 2023). These physical changes have been observed to alter many biological and ecological processes in nature, spanning from the individual level (metabolism, behavior; Conallin et al. 2011), the population and assemblage levels (e.g., community composition; Morrongiello et al. 2011; Datry et al. 2013; Benejam et al. 2016; Huang et al. 2025), and even the whole‐ecosystem level (e.g., altered process rates and fluxes; Colls et al. 2019; Palmer and Ruhí 2019; Rytwinski et al. 2023).

While observational studies in nature show that low flows are associated with the degradation of many aspects of ecosystem structure and function, experimental studies offer an opportunity to explore the mechanisms at play (Hanson and Walker 2020). However, experiments seeking to quantify impacts of low flows on river biota and ecosystem processes have often yielded inconclusive or inconsistent results (White et al. 2022), sometimes even when using the same experimental facility and similar treatments, but different choices in experimental design (e.g., multi‐annual vs. seasonal experiments; Saffarinia et al. 2022, Leathers et al. 2024). This discrepancy begets the question: to what extent have past inferences on low‐flow impacts been biased by unaccounted variation in experimental design and focal ecological endpoints? Answering this question could help improve our understanding of current and projected drought impacts on riverine biodiversity and ecosystem functioning.

Inconsistency in low‐flow responses may arise from differences in experimental design. Indeed, experiments can differ widely in the severity of the drought treatment (e.g., intensity, duration), the extent of replication across space and time, and the scale of the experimental facilities used, which span from laboratory microcosms and mesocosms (Font et al. 2021) to relatively large, open‐air in situ channels (Rosero‐López et al. 2022). In situ experiments tend to be more representative of “real life” conditions (Diamond 1986; Downes et al. 2002), whereas ex situ experiments offer greater replication (thus, statistical power) and control over confounding variables, making them well suited to elucidate mechanisms connecting low flows to dependent variables, and potential interactions among drought‐related stressors. Finding the ideal tradeoff between control and realism is not trivial and often depends on the specific response variable of interest (Ferreira et al. 2023).

Another source of variation relevant to experimental design concerns the type and adequacy of controls. Controls can be spatial, with one site serving as a benchmark for another site that is subjected to flow reduction (Control‐Impact design, Schlief and Mutz 2009); or temporal, where the same site first serves as control and then is subjected to the treatment (Before‐After design, e.g., Baattrup‐Pedersen et al. 2020). However, relatively simple designs with only one comparison tend to underestimate experimental effects (Christie et al. 2019), and conflate treatment effects with effects from confounding variables. These shortcomings led to the development of more robust designs such as BACI (Before‐After–Control‐Impact, Downes et al. 2002), with origins in the “difference in difference” design from econometrics. In a BACI design, at least 2 sites are studied twice, before and after one of them is subjected to the treatment. With BACI designs, the interaction term between space (site) and time (treatment) reveals treatment effects while accounting for shifting baselines as well as systematic variation in the environment (Smith et al. 1993; Chevalier et al. 2019). Recent research has shown the important limitations of space‐for‐time substitution approaches when seeking to quantify climate change impacts on rivers via observational data (Leathers et al. 2025). However, it remains unclear whether the higher complexity and cost of BACI designs in manipulative experiments could be warranted—or whether it would not fundamentally change our understanding of streamflow reduction impacts.

Another important source of variation in the outcomes of flow reduction experiments likely relates to the level of biological organization studied. Responses of individuals and populations can propagate to the higher levels of biological organization (Raffard et al. 2023; Siqueira et al. 2024; Marin et al. 2025), but compensatory dynamics between species can also stabilize the higher levels of organization (e.g., a food web; Kohli et al. 2019; Gianuca et al. 2024; Ma et al. 2025). For instance, when two species compete with each other, a decrease in the biomass of one may be balanced by an increase in the competitor, potentially resulting in no change in overall community biomass (Frost et al. 1995; Morgan Ernest and Brown 2001; Loreau and de Mazancourt 2013). In the same vein, in a functionally‐redundant community a species extirpation may be muted by the gain or presence of species with similar ecological functions (Naeem 1998; Griffin et al. 2009; Biggs et al. 2020). Therefore, changes in structural measures (e.g., community diversity) may not always translate into functional processes or community‐level properties (e.g., total community abundance or biomass). Additionally, some organisms (e.g., “EPT” taxa: mayflies, stoneflies, and caddisflies) tend to be disproportionately sensitive to low flows; and organisms most sensitive to disturbance generally hold high trophic levels, with relatively larger body sizes, longer lifespans, and higher metabolic needs (Voigt et al. 2003; Woodward et al. 2005; Cowell et al. 2025). Determining how drought responses vary within food webs and across levels of biological organization is key to understanding stream ecosystem resilience.

Here, we conducted a meta‐analysis of experimental studies on the impacts of flow reduction in rivers published to date, to assess to what extent our current understanding of low‐flow impacts on river ecosystems results from arbitrary choice related to the experimental design rather than from real ecological dynamics. We investigated the role of experimental design, type of variable, and ecosystem endpoint at which measurements were made, and level of biological organization studied. Our work builds on past research on the topic that focused on methodological or ecological aspects independently (Sabater et al. 2018; Jackson et al. 2016).

We addressed three specific questions: (*Q1) To what extent were past estimates of flow reduction impacts influenced by the design of the experiments developed (*e.g., *controls over space–time; nature of disturbance)?* We expected that simpler designs (i.e., Before‐After and Control‐Impact) might have underestimated low‐flow impacts relative to more complex BACI designs; and we expected stronger effects for relatively more intense flow reductions (Christie et al. 2019; Aspin, Matthews, et al. 2018; Miller et al. 2007). *(Q2) How do flow reduction impacts vary across types of ecological responses measured (i.e., quantity, structure, functioning); and for a given variable across ecosystem endpoint?* We expected that effects on structure (e.g., community richness and diversity) would exceed those on quantity and function (Loreau and de Mazancourt 2013; Biggs et al. 2020); and because species at higher trophic levels tend to be more sensitive (Voigt et al. 2003; Cowell et al. 2025), we expected relatively stronger responses by the higher trophic levels. *(Q3) Do flow reduction impacts vary across levels of biological organization?* We expected that compensatory dynamics and functional redundancy (Naeem 1998; Biggs et al. 2020) would buffer the propagation of lower‐level responses (individuals and populations) to the higher levels of biological organization (communities and whole‐ecosystem). Answering these questions would reveal the unbiased effects of reduced flow on river ecosystem structure and functioning, and could provide valuable guidance for designing future global change experiments in freshwaters.

## 2 Methods

### 2.1 Literature Search, Screening, and Database Building

We conducted a literature search of experimental studies published up to May 2026 that examined the effects of flow reduction on river ecosystems using temporal (Before–After; BA), spatial (Control–Impact; CI), and spatio‐temporal designs (Before–After−Control–Impact; BACI). To identify relevant studies, we searched the Web of Science Core Collection database (Environmental Sciences Ecology, timespan = All years) using the following keywords limited to title and abstract: TS = (“low flow” OR drought) AND TS = (experiment* OR channel OR mesocosm OR artificial* OR microcosm OR flume) AND TS = (stream* OR river* OR lotic) AND TS = (bacteri* OR alga* OR biofilm OR periphyt* OR macrophyt* OR *invertebr* OR arthropod* OR crustace* OR crayfish* OR insect* OR mollus* OR amphibian* OR fish* OR litter OR “organic matter decomposition” OR “leaf breakdown” OR decay OR “primary producti*” OR chlorophyll* OR metabolism OR “gross primary production” OR respiration OR nutri* OR phosphorus OR nitrogen OR “dissolved organic carbon”). We validated our search strategy by confirming that it successfully retrieved four known relevant studies (Truchy et al. 2020; Sabater et al. 2018; Aspin et al. 2018; Walters and Post 2008).

We followed the “Preferred Reporting Items for Systematic Reviews and Meta‐analysis (PRISMA)” method (Liberati et al. 2009; Figure S1) to select relevant articles for our meta‐analysis on the effects of experimental flow reduction on river ecosystems. Specifically, selected studies should (i) be in situ or ex‐situ experiments (modelling and observational studies were excluded); (ii) include temporal comparisons (Before After), spatial comparisons (Control‐Impact, Gradient), and spatio‐temporal comparisons (i.e., Before‐After−Control‐Impact); (iii) focus on the aquatic part of the river system (studies on riparian, hyporheic, or groundwater habitats were excluded); (iv) include mean and standard error or standard deviation values of ecological responses related to animal and plant groups, ecosystem functions and processes (e.g., studies on temperature alone were excluded); and (v) report values for discharge (e.g., in m^3^ s^−1^), or when not available, for velocity (e.g., in m s^−1^) in the controls and treatments. We excluded sediment desiccation experiments because they are only marginally related to flow reduction. Our initial search identified 3295 records, all written in English. We identified 10 relevant reviews, meta‐analyses and book chapters that were manually checked and allowed us to retrieve an additional 13 publications that were not identified via the Web of Science search. All the 3308 publications were manually screened based on title and abstract and a subset of 343 articles were considered suitable for a full‐text review. Based on the eligible criteria previously described, 285 publications were excluded after in‐depth review (e.g., hyporheic or groundwater habitats, desiccation experiments, review papers).

To investigate the potential influence of study design on reported impacts, we coded each study according to its experimental approach: Before–After (BA), Control–Impact (CI), or Before–After– Control–Impact (BACI). For a few studies (i.e., Avery‐Gomm et al. 2014; Espinosa et al. 2020; Liu et al. 2021) the effects of low flows were studied using a gradient of water discharge/velocity. In these studies, the effect sizes were calculated using a CI approach, with the highest flow value serving as “control” and all lower values serving as “impact”. For each article, we recorded the geographical location, species identity and/or major taxonomic group (e.g., fish, amphibians, invertebrates) and the response variables measured (e.g., energy flux; see dataset https://doi.org/10.57745/YMCXH2 for the full list of extracted information). We calculated the percentage reduction in water velocity or discharge, depending on data availability in the original studies, using the formula:

(1) $$\text{Flow reduction}\;\left(\%\right)=\frac{{\text{Flow}}_{\text{Control}}-{\text{Flow}}_{\text{Impact}}}{{\text{Flow}}_{\text{Control}}}\;\times 100$$

where ${\text{Flow}}_{\text{Control}}$ is the discharge or the velocity used in the Control treatment and ${\text{Flow}}_{\text{Impact}}$ is the discharge or the velocity used in the Impact treatment, either in volume/time or in distance/time units.

We then classified studies according to the level of biological organization studied (i.e., individual, population, community, or ecosystem). To improve ecological interpretability, we grouped all measured response variables into three outcome categories: quantity (e.g., abundance, biomass), structure (e.g., diversity), and functioning (e.g., ecological processes; see dataset), while the biological model consisted of seven ecosystem endpoints: (i) water chemistry (all measured physico‐chemical parameters), (ii) detritus (dead organic matter), (iii) microbial decomposers (bacteria, fungi, microbes), (iv) primary producers (algae, amphibious plants, biofilm, macrophytes, periphyton), (v) invertebrates, (vi) vertebrates (fish, amphibians), and (vii) multi‐trophic groups (e.g., food web, river metabolism; Figure S2).

Finally, we extracted statistics for control and treatment groups from tables and results, including sample sizes, means, and measures of variation (standard deviations, standard errors, or confidence intervals). When necessary, we extracted data from figures using the WebPlotDigitizer software (v.3.4; Rohatgi 2015) or the R package metaDigitise (v1.0.1; Pick et al. 2019). If key information (e.g., velocity or discharge, means and SD/SE values of the response variables) were not available for a specific study, we contacted the corresponding author. The final data set included 53 relevant articles, for a total of 1284 effect sizes grouped in 1082 studies. We defined a “study” according to the methodology proposed by Nakagawa, Yang, et al. (2023), combining several effect sizes derived from the same control group, especially in BACI designs with repeated *After‐impact* measurements. One article may therefore contain several studies.

### 2.2 Data Analysis

We calculated effect sizes using the standardized mean difference method, assuming Gaussian distributions and following Christie et al. (2019). We then applied a correction for small sample sizes using the Hedges'* d* method (Borenstein 2009).

For several response variables, specifying the expected direction of the effect is subjective and difficult, which in turn can bias the assessment of the strength and direction of the impact. For this reason, we chose to use the absolute values of effect sizes (|Hedges' *d*|), as they represent the magnitude of the impact regardless of its direction.

The multilevel meta‐analysis model was fitted using the rma.mv function in the metafor R package (Viechtbauer 2010), and included random intercepts for study identity (*StudyID*) and for individual effect size (*ID*) to account for the non‐independence of multiple effect sizes from the same study. Within‐study correlations among sampling errors were modelled by imputing a variance–covariance matrix with an assumed correlation of 0.8 (Williams et al. 2025). Similar results were obtained with correlations of 0.5 and 0.9, indicating robustness to this assumption (Table S1).

Effect sizes from studies with fewer replicates tend to have higher uncertainty, and often show larger treatment effects than those from larger sample sizes (Nakagawa, Yang, et al. 2023). To assess and adjust for potential small‐study bias, we further included the moderator √(1/𝑛̃), where 𝑛̃ represents the effective sample size of each effect size (Nakagawa, Yang, et al. 2023). This variable captures the expected association between sampling error and effect size magnitude, providing a model‐based correction for small‐study effects. Additionally, time‐lag bias can occur if inconclusive results take longer to be published than positive test results. Thus, effect sizes sometimes tend to approach zero over time, a pattern known as ‘declining effects’ (Costello and Fox 2022). To test this potential source of bias, we included centered publication years as a moderator in the meta‐analytic model. Heterogeneity represents the variation between effect sizes, which is not accounted for by the sampling error variance. To quantify it in our meta‐analysis, we used the R package orchaRd (Nakagawa, Lagisz, et al. 2023). It calculates the percentage of variation among effect sizes not explained by sampling error, and the heterogeneity explained by each additional random effect in the model (i.e., between‐ and within‐study effect). We then conducted a sensitivity analysis using a leave‐one‐out approach, which involves performing multiple meta‐analyses, each time excluding one study. This method allowed us to assess the influence of each study on the overall estimate of effect size, and to identify studies with an exaggerated influence on the results.

To study differences in the magnitude of effect sizes among experimental designs, we included the design type, that is, Before‐After (BA), Control‐Impact (CI), and Before‐After−Control‐Impact (BACI), as a fixed effect in the meta‐analytic model. We also included the intensity of flow reduction (logit transformed) and the duration of low‐flow periods (log transformed) as interaction terms with the design in the meta‐regression model. Finally, because the different categories of biological organization levels, ecological responses, and ecosystem endpoints were not evenly distributed across the designs, we decided to create three sub‐datasets filtering for each design. We then used our meta‐analysis model for each sub‐dataset, adding the organization level, ecological response level, or ecosystem endpoint as a fixed effect. We then created a model for each study design. Because |Hedges' *d*| values were right‐skewed and included zeros, we applied a log transformation with a small offset: log(|*d*| + 0.5) for the meta‐regression models (Table S2). Sampling variances on the log scale were approximated using the delta method (Dorfman 1938). Below, we reported the coefficients of regression (*ß*, log‐scaled) for each meta‐regression model including confidence interval (CI_95%_) and *p*‐values, where a positive *ß* means a positive relationship between the tested variable and effect size values. We also reported the mean effect size (ES) for models using categorical variables (e.g., design, ecological response, ecosystem endpoint, biological organization).

## 3 Results

### 3.1 Overview of Studies Included in Our Meta‐Analysis

**FIGURE 1:**
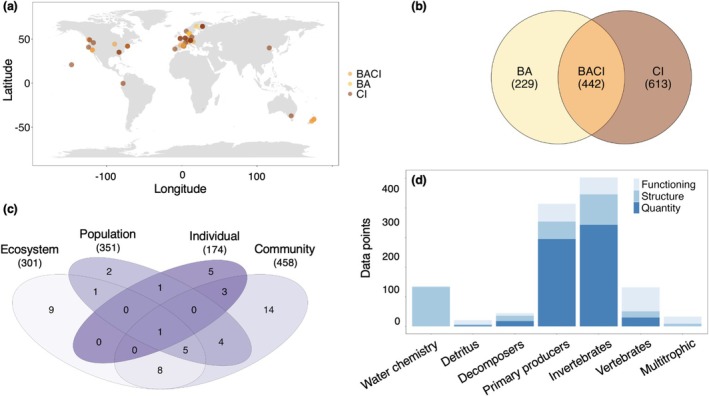
Universe of studies considered, and coverage across key geographic, experimental, and ecological dimensions. (a) Mapping of articles used for the meta‐analysis, colored according to the experimental design used. (b) Schematic representation of the effect size percentages obtained for each design. The number of effect sizes considered is shown in parentheses. (c) Venn diagram showing the number of articles at each level of biological organization, and at the intersection of multiple levels of organization. The number of effect sizes considered is shown in parentheses. (d) Number of effect sizes per ecosystem endpoint, color‐coded by type of response measured (quantity, structure, functioning).

A total of 53 individual experiments reporting 1284 effect sizes (or responses to flow reduction) were collated, mainly distributed across Europe (63%), North America (17%), Oceania (14%), Asia (5%), and South America (1%) (Figure 1a). Noting this heterogeneity, we first sought to map and examine geographical patterns in ecological responses to drought (Figure S3).

Most of these effect sizes (82%) included a control site, either as part of a CI or a BACI design (Figure 1b). However, only 34% of the effect sizes included both spatial and temporal replicates. Studies using a temporal control, such as in BA design, represented only 18% of the total effect sizes.

Experimental reductions in discharge (or flow velocity) ranged from 33% to 100%, with a median of 88% (84% for velocity). The duration of flow reduction treatments ranged from 15 min to 2 years, with a median of 1 month. Studies focused mainly on the community level (36% of the effect sizes), followed by population (27%) and ecosystem levels (23%). In contrast, individual‐level studies were much rarer (14%). In total, 43% of the articles spanned two or more levels of organization. For example, most ecosystem‐level studies also included a community or a population‐level endpoint (Figure 1c). In contrast, individual‐level studies rarely included other levels of biological organization.

Regarding the type of ecological response, quantity metrics were most commonly assessed (54% of the effect sizes distributed across 39 articles), followed by metrics of structure (27% from 25 articles), and of functioning (19% from 24 articles). The most represented ecosystem endpoints were invertebrates (39%) and primary producers (32%), followed by water chemistry (11%—mostly nutrients measures), and vertebrates (10%). Decomposers (4%) and detritus (2%) were reported rarely, and studies spanning multitrophic interactions were also rare (2%) (Figure 1d). Different categories were typically studied for different endpoints. For instance, measures related to structure (e.g., diversity) were often assessed for primary producers and invertebrates (46% of the structure effect sizes).

### 3.2 Bias and Sensitivity Analysis

While we found no bias related to study size, √(1/𝑛̃) (*ß* = 0.064, CI_95%_ = [−0.108; 0.236], *p* = 0.464; QE_(df=1282)_ = 6027.144, *p* < 0.001; Figure S4), study size and experimental design are strongly confounded. BACI designs display the highest effective sample sizes (median *ñ* = 40.9) while BA designs the lowest (*ñ* = 2.0) with near‐zero variance, precluding reliable estimation of design‐specific small‐study effects. We therefore account for study size as a covariate in the pooled model rather than estimating design‐specific effects. Moreover, we found no ‘declining effect’, or bias related to the year of publication (*ß* = −0.003, CI_95%_ = [−0.009; 0.004], *p* = 0.427; QE_(df=1282)_ = 6097.208, *p* < 0.001; Figure S5). Total heterogeneity was 66%, of which 46% was due to between‐study variance and 20% to within‐study variance. The heterogeneity values from all the meta‐analysis models presented in this study are available in Table S3. A sensitivity analysis revealed that the meta‐analytic model used was robust, and that its results were not influenced by any particular study.

### 3.3 Objective 1: Effects of Experimental Design

**FIGURE 2:**
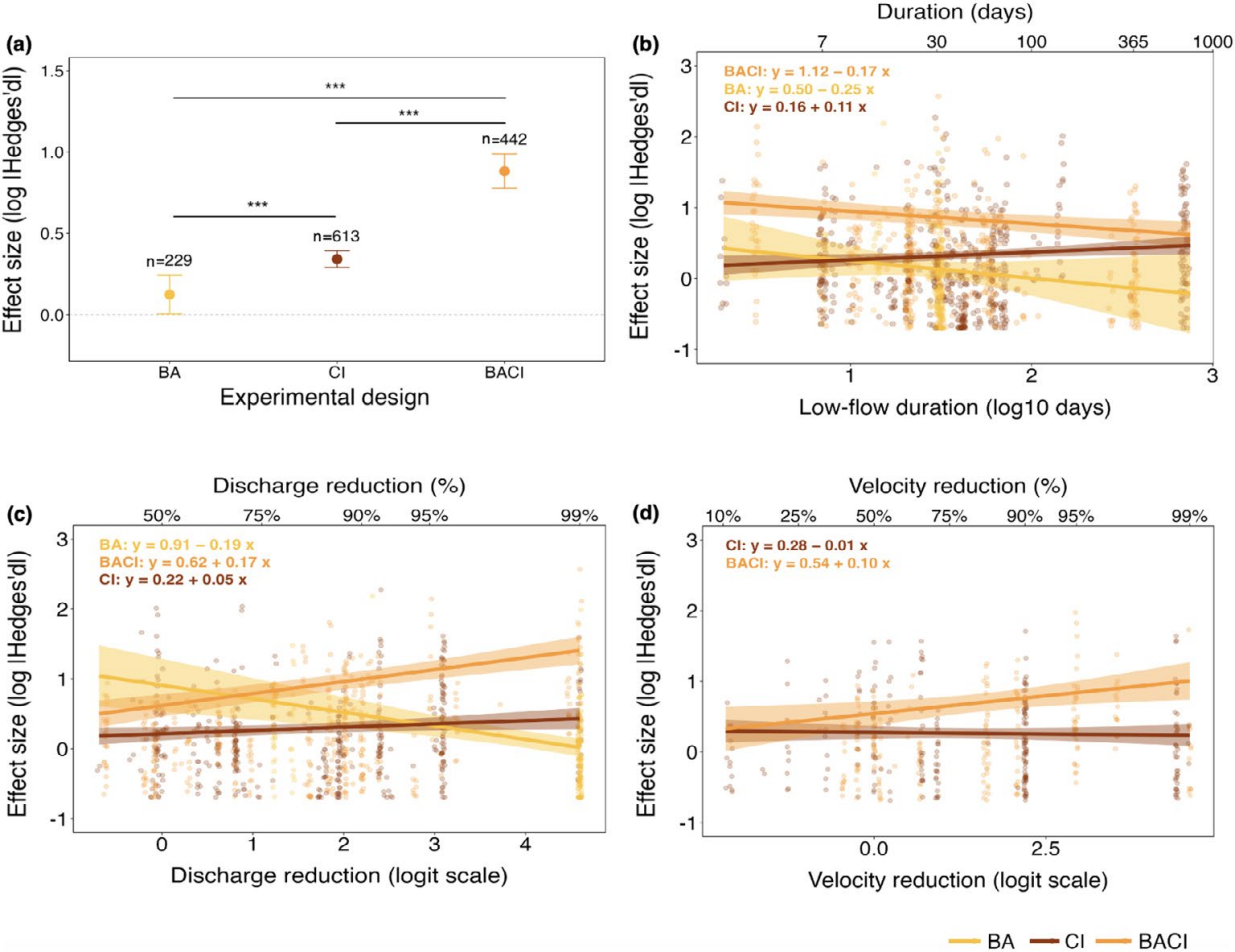
Relationships between major experimental choices and measured low‐flow effects. (a) Magnitude of log |Hedges' *d*| for BA, BACI, and CI designs. *n* indicates the number of effect sizes for each design. (b–d) Relationships between the different experimental designs (BA, CI, BACI) and effect sizes (log |Hedges' *d*|) across different dimensions of flow reduction: (b) Duration of the low‐flow period, measured as log_10_(day); (c) Magnitude of discharge reduction, measured as logit (percentage of discharge reduction); and (d) Magnitude of water velocity reduction, measured as logit (percentage of water velocity reduction).

When examining the effects of experimental design (Q1), we found that BACI designs yielded significantly larger effect sizes than both BA designs and CI designs (Figure 2a). After SSE correction, estimated effect sizes (log‐magnitude scale) were: BACI = 0.882 [0.777; 0.988], BA = 0.124 [0.004; 0.244], and CI = 0.342 [0.290; 0.394]. The BA estimate decreased by 59% relative to the uncorrected estimate (BA = 0.301 [0.193, 0.410] without SSE correction). The BACI estimate increased after correction, consistent with larger, better‐replicated studies resolving stronger drought signals. The CI estimate remained comparatively stable.

After accounting for this bias, the BACI–BA contrast corresponds to an approximately 2.1‐fold difference in estimated effect‐size magnitude (back‐transformed log scale, offset included), while the BACI–CI contrast corresponds to a 1.7‐fold difference. Together, these results underscore the substantive inferential consequences of study design choice in drought impact assessment.

Despite BA and CI designs being the most common (66% of the effect sizes), they have likely been underestimating impacts of low flows. Note that residual heterogeneity remained substantial (QE_(df=1280)_ = 4913.4, *p* < 0.0001), with total *I* ^2^ = 63.1%, partitioned between study‐level (I^2^_StudyID_ = 44.1%) and observation‐level (I^2^_ID_ = 19.1%) variance, indicating that unmeasured factors contribute substantially to variability beyond experimental design.

We found no significant effects of low‐flow duration. The magnitude of ecological responses did not change over time with flow reduction, regardless of the design employed (*ß* = −0.022, CI_95%_ = [−0.092; 0.049]; *p* = 0.543; QE_(df=1185)_ = 5052.401; *p* < 0.0001; Figure 2b). However, the relationship between low‐flow duration and effect size did differ significantly between BACI and CI designs, with longer low flows reducing effects under BACI designs, but increasing effects under CI designs (*ß*_BACI_ = −0.175, CI_95%_ = [−0.2823; −0.068]; *ß*_CI_ = 0.123, CI_95%_ = [−0.127; 0.372]; *p*_BACI‐CI_ < 0.0001).

The relationship between the magnitude of discharge reduction and effect size also depended on study design (Figure 2c). For BA studies, more severe discharge reductions were associated with smaller effect sizes (*ß* = −0.194, CI_95%_ = [−0.283; −0.105]; *p* < 0.0001), while for BACI and CI studies, stronger flow reductions increased effect sizes (*ß*_BACI_ = 0.171, CI_95%_ = [−0.032; 0.311]; *p*_BA‐BACI_ < 0.0001 and *ß*_CI_ = 0.047, CI_95%_ = [−0.087; 0.180]; *p*_BA‐CI_ < 0.0001). A significant interaction revealed that BACI studies tended to capture larger effects as reductions in water velocity became more severe (*ß* = 0.101, CI_95%_ = [−0.035; 0.219]; *p* = 0.013; Figure 2d), while CI studies did not (*ß* = −0.009, CI_95%_ = [−0.049; 0.032]). These results suggest that BACI designs were more sensitive to severe low‐flow treatments.

### 3.4 Objective 2: Effects of Response Types and Ecosystem Endpoint

**FIGURE 3:**
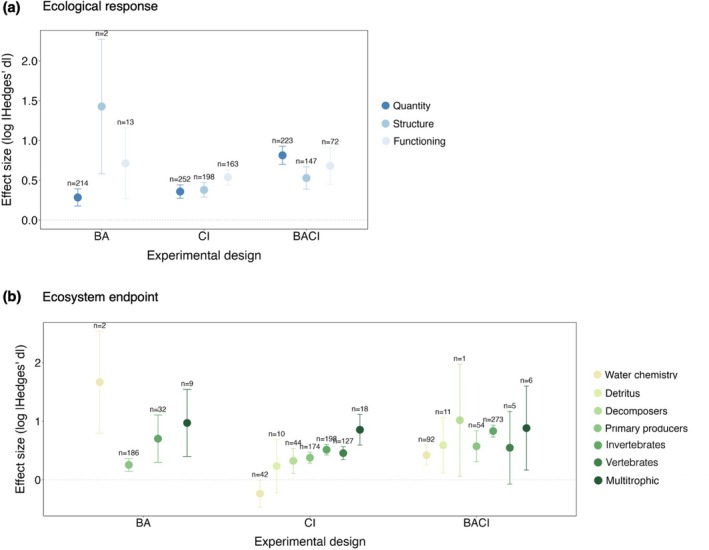
Relationships between types of ecological endpoints monitored and measured low‐flow effects. Scatter plots showing estimated effect sizes (log |Hedges' *d*|) and corresponding 95% confidence intervals by study design and (a) type of ecological response measured, or (b) ecosystem endpoint. The number of effect sizes for each category is shown above each confidence interval.

Measures of quantity, structure, and functioning did not differ in how they responded to low flows (ES_Quantity_ = 0.444, CI_95%_ = [0.382; 0.507]; ES_Structure_ = 0.377, CI_95%_ = [0.298; 0.456]; ES_Functioning_ = 0.438, CI_95%_ = [0.351; 0.524]; QE_df=1281_ = 5803.984, *p* < 0.0001). In addition, we found no significant difference between the type of response measured for CIs (*p* > 0.05; Figure 3a). However, for BACI designs, effect sizes for structure measurements were significantly lower than for quantity measurements (ES_Structure_ = 0.528, CI_95%_ = [0.388; 0.667] and ES_Quantity_ = 0.814, CI_95%_ = [0.700; 0.927]; *p* = 0.002), whereas the opposite was found for studies using BA design (ES_Structure_ = 1.427, CI_95%_ = [0.582; 2.272] and ES_Quantity_ = 0.284, CI_95%_ = [0.177; 0.392]; *p* = 0.008).

We found significant differences in low‐flow responses between ecosystem endpoints from studies using BA, CI, and BACI designs (*p* < 0.05; Figure 3b). More specifically, for all designs, effect sizes tended to increase from lower to higher trophic positions. For BA designs, even if mean effect size for water chemistry was higher than for other ecosystem endpoints (ES = 1.666, CI_95%_ = [0.796; 2.536], *p* < 0.001), multitrophic and invertebrates effect sizes were significantly higher than primary producers effect size (ES_Multitrophic_ = 0.970, CI_95%_ = [0.396; 1.544]; ES_Primary producers_ = 0.254, CI_95%_ = [0.143; 0.364], *p*_Primary producers‐Multitrophic_ = 0.016; ES_Invertebrates_ = 0.700, CI_95%_ = [0.295; 1.105], *p*_Primary producers‐Invertebrates_ = 0.039). The same pattern was also observed for CI, with a tendency for effect sizes to increase by 18% on average with trophic level and, to a lesser extent, for BACIs, with a tendency for effect sizes to increase by 7% on average with trophic position, with a high variability. In general, we also observed narrower confidence intervals for CIs than for other designs, likely reflecting larger sample sizes (Figure 3).

### 3.5 Objective 3: Effects of Levels of Biological Organization

**FIGURE 4:**
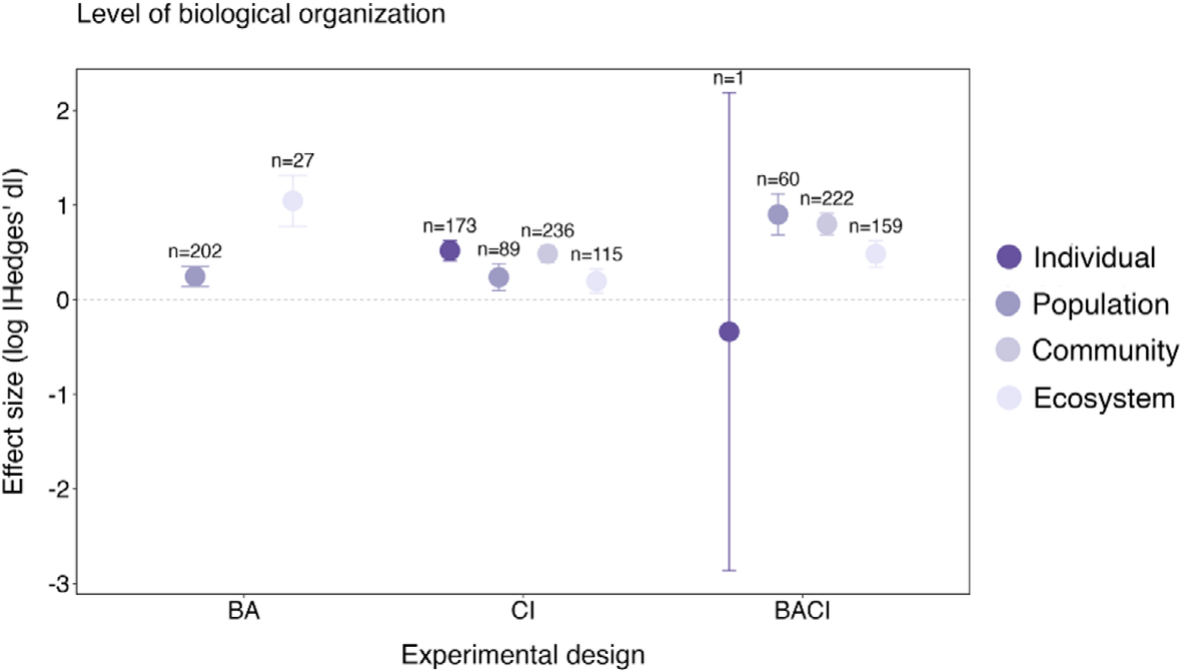
Relationship between level of biological organization chosen and measured low‐flow effects. Scatter plots showing effect size estimates (log |Hedges' *d*|) and their corresponding 95% confidence interval by study design and level of biological organization. The number of effect sizes for each category is shown above each confidence interval.

Low‐flow responses at the community level (ES = 0.627, CI_95%_ = [0.557; 0.698]) were generally stronger than responses observed at the ecosystem level (ES = 0.356, CI_95%_ = [0.262; 0.449], *p* < 0.0001), individual level (ES = 0.330, CI_95%_ = [0.243; 0.418], *p* < 0.0001) or population level (ES = 0.309, CI_95%_ = [0.222; 0.396], *p* < 0.0001; Figure 4). However, experimental design mediated these relationships. For BA designs, effect sizes were larger at the ecosystem than at the population level, although we caution that the latter had one of the lowest sample sizes of all categories (ES_Ecosystem_ = 1.045, CI_95%_ = [0.774; 1.315], *p* < 0.0001; ES_Population_ = 0.247, CI_95%_ = [0.141; 0.353]). In turn, effect sizes in CI and BACI designs tended to decrease while moving up in levels of biological organization, as we initially hypothesized. Indeed, for CI designs, effects at the ecosystem level (ES = 0.196, CI_95%_ = [0.068; 0.324]) were about 2.5× smaller than at the community level (ES = 0.485, CI_95%_ = [0.395; 0.575], *p =* 0.001). In the same way, differences in low‐flow effects across levels of biological organization under BACI designs showed the same pattern of decreased effect sizes with increasing level of biological organization, except for the individual level for which the effect size is smaller, but for which we have only a single value (ES_Individual_ = −0.337, CI_95%_ = [−2.862; 2.189]; ES_Ecosystem_ = 0.484, CI_95%_ = [0.345; 0.622], ES_Community_ = 0.800, CI_95%_ = [0.687; 0.913], *p*_Community‐Ecosystem_ = 0.001; and ES_Population_ = 0.901, CI_95%_ = [0.684; 1.119], *p*_Community‐Population_ = 0.002). Overall, the BACI and CI designs converged in showing some buffering of low‐flow impacts at the highest levels of biological organization (Figure 4).

## 4 Discussion

A river's flow regime is considered a ‘master’ variable driving physical structure and ecological functioning (Poff et al. 1997; Palmer and Ruhí 2019), but experiments testing the sensitivity of different ecological endpoints to low‐flow reductions have often yielded inconsistent results (e.g., Saffarinia et al. 2022; Leathers et al. 2024). Given the need to predict ecological responses to future reductions in flows, we sought to resolve these past inconsistencies by developing a meta‐analysis that examined how variation in study design, ecological responses measured, and levels of biological organization could have driven variability in drought impact estimates. We found that the type of experimental design strongly influenced the detectability and magnitude of drought impact estimates, with BACI studies detecting 2.1× larger impacts than BA and 1.7× larger impacts than CI‐type studies. Responses varied across experimental design types, but tended to be stronger at the higher trophic levels; and tended to be buffered at the higher levels of biological organization, in agreement with the expected benefits of compensatory dynamics (Kohli et al. 2019; Ma et al. 2025) and functional redundancy (Naeem 1998; Griffin et al. 2009; Biggs et al. 2020). Our study highlights that the existing literature is overwhelmed by weak experimental designs (68% of the papers lack spatio‐temporal controls) that vastly underestimate flow reduction impacts. Stronger designs, and studies spanning multiple levels of biological organization, would improve our mechanistic understanding of drought impacts—a key need given the widespread reductions in river flows projected under climate change (Satoh et al. 2022).

### 4.1 The Importance of Spatio‐Temporal Monitoring and Controls

Our study shows that experimental designs with proper spatio‐temporal controls (i.e., BACI designs) are needed to adequately quantify flow reduction impacts. This finding aligns with Christie et al. (2019), who simulated ecological disturbance and demonstrated that BACI outperformed BA and CI designs in detecting both the correct direction and magnitude of effects. Specifically, BA and CI correctly detected the direction of the effects 70% of the time (against 100% for BACI), and underestimated their magnitude 55% of the time. In our meta‐analysis we used absolute values of effect sizes, so the tendency of BA and CI to misidentify the direction of the effects did not affect our results. However, the tendency to underestimate effect sizes likely explains variation across past experimental studies, which are largely dominated by BA and CI designs.

The poor performance of non‐BACI designs likely stems from differences in response variables being driven by factors other than the applied disturbance, temporally for BA designs (e.g., variations due to seasonal changes) and spatially for CI designs (e.g., when the control site is already different from the impact one before the flow reduction). Such biases may be more concerning in observational than in experimental studies, as in the former very few external sources of variation can be controlled (e.g., in ‘space‐for‐time substitution’ studies; Leathers et al. 2025). Our results reveal that design‐level limitations interact with a systematic precision deficit. In particular, BA and CI studies in this literature are conducted with substantially smaller effective sample sizes than BACI studies, generating a small‐study bias that inflates effect size estimates. After correcting for this bias, BA effect sizes decrease markedly (uncorrected: 0.301; bias‐corrected: 0.124), whereas BACI estimates increase (0.663–0.882), consistent with improved identification under stronger spatio‐temporal control. This pattern indicates that a literature dominated by BA and CI designs (68% of studies; 66% of effect sizes) yields estimates that are both directionally less reliable (Christie et al. 2019) and systematically inflated in magnitude relative to what low‐replication designs can support. More broadly, non‐BACI designs produce effect size estimates that are simultaneously inflated relative to their bias‐corrected counterparts and attenuated relative to BACI, reflecting the joint influence of small‐study effects and incomplete spatio‐temporal control. However, our primary magnitude metric, log(|*d*| + *c*), is known to be upward‐biased and variance‐compressing, which likely attenuates between‐group differences in absolute effect size.

### 4.2 Bridging Extreme Events to Shifting Regimes

The severity of flow reduction appeared to have a significant positive effect on the magnitude of ecological responses, especially in BACI and CI‐type studies, but contrary to our hypothesis, low‐flow duration did not explain variation in effect sizes. No negative correlation was found between discharge reduction and duration across studies (Figure S6), suggesting that this pattern is not an artifact driven by the universe of studies included in our meta‐analysis, but rather likely reflects real ecological dynamics. Specifically, we believe it could be driven by a combination of acclimation of the organisms that were already present at the beginning of the experiment (Colls et al. 2019; Lake 2011), and the replacement of drought‐sensitive organisms with more drought‐tolerant ones. Community replacement (or turnover) has been observed in the longest experiments included in our meta‐analysis (two years; Ledger et al. 2013); as well as in multi‐year studies conducted in terrestrial ecosystems (e.g., Ohlert et al. 2025). Community turnover, in the form of species reordering (i.e., changes in relative abundances), as well as gains and losses, has also been identified as a major mechanism connecting riverine drought to ecological change in long‐term field studies (Leathers et al. 2025). However, the majority of the studies included in our meta‐analysis lasted fewer than 100 days (86% of the effect sizes). These findings should therefore be interpreted with caution, as the absence of a relationship between drought duration and effect sizes may also reflect the limited availability of long‐term studies.

Additionally, the drastic flow reductions imposed in the considered experiments may have led to immediate rather than delayed or gradual ecological responses. Indeed, the magnitude of flow reductions observed in experiments was very severe, that is, 88% for discharge and 84% for velocity (medians), compared to ca. 50% reduction in annual mean low flow under various scenarios of climate change (Nohara et al. 2006; van Vliet et al. 2013). However, the severity of these events may not be unrealistic from extreme episodes that are increasingly observed in anomalously dry years. Indeed, severe drought events, often leading to “novel” flow intermittence, are increasing in intensity, duration, and spatial extent globally (Carlson et al. 2024; Mimeau et al. 2025). Moreover, these different facets of drought and drying can interact to impact ecosystems in the long term (Frank et al. 2015; Miller et al. 2011; Ummenhofer and Meehl 2017) in ways that experiments would not necessarily capture. For instance, impacts of extreme low flows can be exacerbated by the spatial extent of drought, as habitat fragmentation hinders post‐drought population and community recovery, increasing watershed‐scale extinction risk (Fournier et al. 2023; Sarremejane et al. 2021). Similarly, although some experiments included in our meta‐analysis lasted for up to two years (Currinder et al. 2014; Ledger et al. 2013), the span of most studies remains too short to address the long‐term impacts of extreme climatic events. Long‐term experiments that examine the different spatio‐temporal facets of extreme events factorially (e.g., severity, duration, extent; Jentsch et al. 2007; Smith 2011), repeating event sequences to understand the effects of shifting regimes, are key next steps to enhance realism and mechanistic insight.

### 4.3 Ecological Complexity Buffers Drought Impacts

Because species at higher trophic levels tend to be larger, longer‐lived, more sensitive to biotic stress (e.g., from the lower food‐web levels) and also to abiotic stress (Petchey et al. 1999; Purvis et al. 2000; Voigt et al. 2003; Hu et al. 2022), we predicted greater effects of flow reduction on the higher trophic levels. Results were consistent with our prediction, where groups of organisms belonging to higher trophic levels were more affected by flow reduction than groups at lower levels. However, we note that the different trophic groups were unbalanced in terms of sample size, experimental designs, and types of responses measured (Figure 3). In turn, in agreement with our third hypothesis, we did find that impacts of flow reduction generally weakened as the level of biological organization increased. This expectation supported the prediction that compensatory dynamics and functional redundancy would buffer responses of individuals and populations. We note, however, that heterogeneity in experimental designs and endpoints also likely reduced our capacity to robustly examine this effect. For instance, studies on the impact of flow reduction at the individual level were scarce, only one used a BACI design, and rarely addressed the possible consequences at higher levels of organization. More generally, no study addressed the impact of reduced flow at more than two levels simultaneously.

These patterns highlight the need for future studies to precisely quantify how the impact of a stressor, such as streamflow reduction, declines along a gradient of ecological complexity, potentially identifying factors that exacerbate or dampen the buffering (Mayor et al. 2024; Tilman 1996). This is important because compensatory dynamics and functional diversity (or “response diversity”) underlie biodiversity‐stability relationships that have been extensively documented across numerous ecosystems (Deng 2012; Gianuca et al. 2024; Hatton et al. 2024; Isbell et al. 2015; Liu et al. 2024; Mahaut et al. 2025; Yachi and Loreau 1999), often underpinning biodiversity conservation strategies. Future experimental studies should also increase realism by evaluating how low flows influence ecological dynamics not only via direct forces (e.g., by altering organismal fitness as a consequence of modifying the physical environment) but also indirectly, by altering competitive, bottom‐up, and top‐down interactions within the food web (Evangelista et al. 2025; Gilman et al. 2010; Jones et al. 1998; Zarnetske et al. 2012).

## 5 Concluding Remarks

Ecologists increasingly rely on manipulative experiments to test predictions and causal pathways explaining ecosystem responses to global change stressors—including water scarcity, warming, nutrient inputs, and biodiversity loss (De Boeck et al. 2015; Hanson and Walker 2020). In turn, inferences from experiments are increasingly incorporated in process‐based ecosystem models. By carefully examining and re‐analyzing the literature on the topic, we found that weak experimental designs lacking controls over space *and* time are widespread, and have led to a severe underestimation of drought impacts on a range of variables of stream ecosystem structure and functioning. We caution that drought impacts are both stronger and more certain than most experiments have previously suggested (Figure 2a). This finding resolves previous inconsistencies, and underscores the need for future studies to adopt BACI designs. However, we acknowledge that implementing experiments based on BACI designs can be challenging (Stewart‐Oaten et al. 1986; Underwood 1994). Specifically, they require extended post‐treatment monitoring periods and are consequently more complex to establish, particularly in field contexts. Moreover, although the dual spatial and temporal controls embedded in BACI designs enhance the capacity to detect drought effects, it cannot be excluded that certain factors, such as experimental scale (ex situ vs. in situ) or the type of experimental facilities, systematically differ between BACI and non‐BACI studies and thereby contribute additional, unaccounted‐for confounding effects (Figure S7). We also contend that future studies should carefully consider both the intensity and duration of flow reductions, exploring less drastic, but longer‐term flow reductions. As hydrologic droughts continue to intensify with projected climate change and widespread freshwater resource overuse (Satoh et al. 2022), the need for robust science to identify ecological thresholds and inform sustainable freshwater resource management is more critical than ever.

## Author Contributions

**Mathieu Buoro:** conceptualization, funding acquisition, investigation, methodology, project administration, supervision, writing – review and editing. **Arturo Elosegi:** conceptualization, funding acquisition, investigation, methodology, project administration, writing – review and editing. **Charlotte Evangelista:** conceptualization, data curation, formal analysis, investigation, methodology, supervision, visualization, writing – original draft, writing – review and editing. **Albert Ruhí:** conceptualization, funding acquisition, investigation, methodology, project administration, supervision, writing – original draft, writing – review and editing. **Eva Haristoy:** conceptualization, data curation, formal analysis, investigation, methodology, visualization, writing – original draft. **Stephanie M. Carlson:** conceptualization, funding acquisition, investigation, methodology, project administration, writing – review and editing. **Cédric Tentelier:** conceptualization, funding acquisition, investigation, methodology, project administration, writing – review and editing.

## Funding

This work was supported by the International Associated Laboratory MacLife and benefited from the support received by E2S‐UPPA, and the Adour‐Garonne Water Agency AID‐2023‐03145. Albert Ruhí was additionally supported by the U.S. National Science Foundation DEB‐2047324.

## Conflicts of Interest

The authors declare no conflicts of interest.

## Supporting information


**Data S1:** Dataset.
**Data S2:** Drought_meta_analysis_script.
**Data S3:** README.
**Figure S1:** PRISMA diagram showing the different steps of the meta‐analysis: studies identification, screening, eligibility and inclusion of papers.
**Figure S2:** Schematic representation of the relationships between the variables used in the meta‐analysis. Shades of purple refer to the levels of biological organization, shades of blue to the type of ecological responses measured, and shades of green to the different ecosystem endpoints.
**Table S1:** Robustness of the Before‐After (BA) vs. BACI design contrast across assumed within‐study correlations (*ρ* = 0–0.9), with model‐based and cluster‐robust (CR2) standard errors.
**Table S2:** Sensitivity analysis of the offset used in the meta‐analysis models.
**Figure S3:** Relationship between climate areas of studies and measured low‐flow effects. Plot showing Effect size estimates (log |Hedges' *d*|) and their corresponding 95% confidence interval by study design and climate area. The number of Effect sizes for each category is shown above each confidence interval.
**Figure S4:** (a) Bubble plot showing the relationship between the effect sizes, calculated as the log |Hedges' *d*| and the adjusted sampling error, measured as √(1/𝑛̃), where 𝑛̃ represents the effective sample size of each effect size. (b) Table listing the values of the small studies effect (√(1/𝑛̃)) for all the models used. Significant *p*‐values are written in bold.
**Figure S5:** Bubble plot showing the relationship between the effect sizes, calculated as the log |Hedges' *d*| and the mean‐centred publication year.
**Table S3:** Table showing the values of total, between and within studies heterogeneity (%) for each meta‐analysis model.
**Figure S6:** Scatter plot showing relationships between the duration of the low‐flow period, measured as log_10_(day) and magnitude of discharge reduction, measured as logit (percentage of discharge reduction).
**Figure S7:** Heatmap showing the number of effect sizes in different categories of level of (a) biological organization and (b) experimental facilities for BA, CI and BACI studies.

## Data Availability

The data presented in this work are available in Supporting Information. In addition, the dataset is available at https://doi.org/10.57745/YMCXH2.
