# Supplementary material for: Widespread Underestimation of Drought Impacts on River Ecosystems Due to Weak Experimental Designs
Source: Glob Chang Biol. 2026 Jul 24;32(7):e70993. doi: 10.1111/gcb.70993 (PMC13397043; doi:10.1111/gcb.70993)
Supplement: Supplementary file 1 — Data S1: Dataset. Data S2: Drought_meta_analysis_script. Data S3: README. Figure S1: PRISMA diagram showing the different steps of the meta‐analysis: studies identification, screening, eligibility and inclusion of papers. Figure S2: Schematic representation of the relationships between the variables used in the meta‐analysis. Shades of purple refer to the levels of biological organization, shades of blue to the type of ecological responses measured, and shades of green to the different ecosystem endpoints. Table S1: Robustness of the Before‐After (BA) vs. BACI design contrast across assumed within‐study correlations (ρ = 0–0.9), with model‐based and cluster‐robust (CR2) standard errors. Table S2: Sensitivity analysis of the offset used in the meta‐analysis models. Figure S3: Relationship between climate areas of studies and measured low‐flow effects. Plot showing Effect size estimates (log |Hedges' d|) and their corresponding 95% confidence interval by study design and climate area. The number of Effect sizes for each category is shown above each confidence interval. Figure S4: (a) Bubble plot showing the relationship between the effect sizes, calculated as the log |Hedges' d| and the adjusted sampling error, measured as √(1/𝑛̃), where 𝑛̃ represents the effective sample size of each effect size. (b) Table listing the values of the small studies effect (√(1/𝑛̃)) for all the models used. Significant p‐values are written in bold. Figure S5: Bubble plot showing the relationship between the effect sizes, calculated as the log |Hedges' d| and the mean‐centred publication year. Table S3: Table showing the values of total, between and within studies heterogeneity (%) for each meta‐analysis model. Figure S6: Scatter plot showing relationships between the duration of the low‐flow period, measured as log10(day) and magnitude of discharge reduction, measured as logit (percentage of discharge reduction). Figure S7: Heatmap showing the number of effect sizes in different categori [file GCB-32-e70993-s001.zip › gcb70993-sup-0001-supinfo/gcb70993-sup-0004-FigureS1-S7-TableS1-S3.docx]

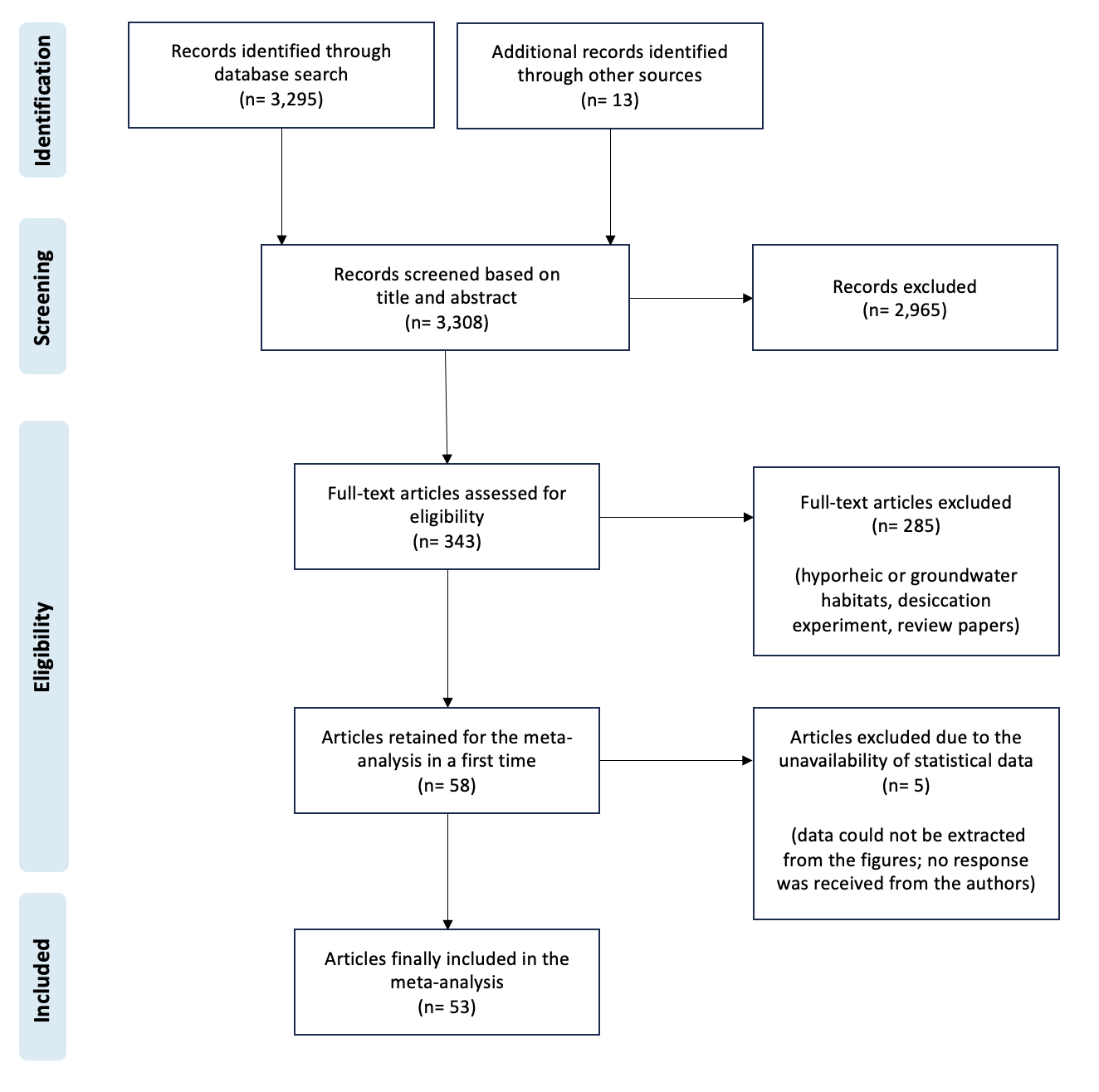


**Figure S1.** PRISMA diagram showing the different steps of the meta-analysis: studies identification, screening, eligibility and inclusion of papers. Our research was conducted in two phases: the first in July 2021 and the second in May 2026. For clarity, we have separated the results of the two studies on the diagram.


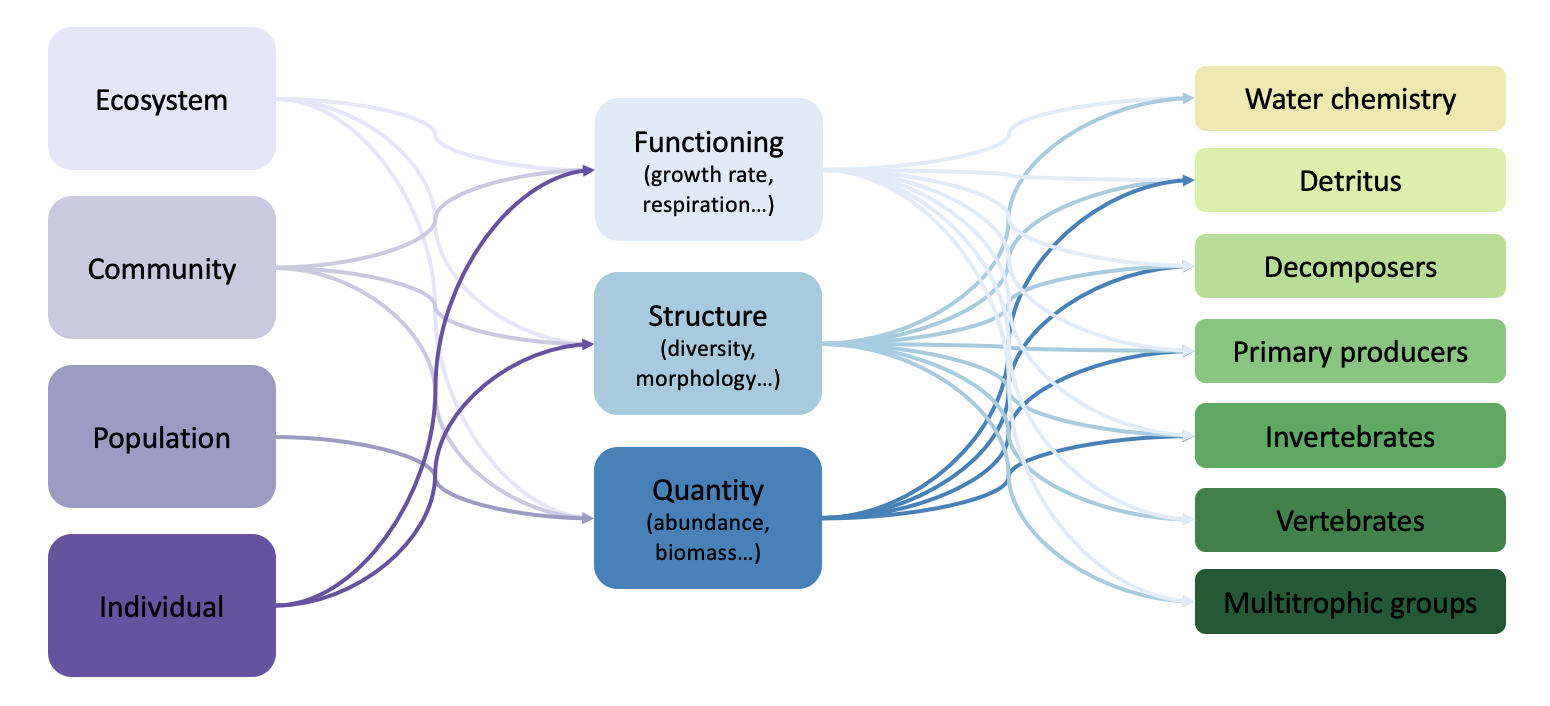


**Figure S2.** Schematic representation of the relationships between the variables used in the meta-analysis. Shades of purple refer to the levels of biological organization, shades of blue to the type of ecological responses measured, and shades of green to the different trophic levels.

**Table S1:** Robustness of the Before-After (BA) vs BACI design contrast across assumed within-study correlations (ρ = 0–0.9), with model-based and cluster-robust (CR2) standard errors.

| ρ | β (BA vs BACI) | SE | SE (CR2) | p | σ²(ES) | logLik |
| --- | --- | --- | --- | --- | --- | --- |
| 0.0 | −0.25 | 0.069 | 0.064 | <0.001 | 0.071 | −1283.7 |
| 0.2 | −0.28 | 0.070 | 0.065 | <0.001 | 0.071 | −1274.3 |
| 0.5 | −0.32 | 0.071 | 0.066 | <0.001 | 0.070 | −1256.6 |
| 0.8 | −0.36 | 0.071 | 0.066 | <0.001 | 0.066 | −1227.2 |
| 0.9 | −0.39 | 0.071 | 0.067 | <0.001 | 0.061 | −1209.9 |

**Table S2**. Sensitivity analysis of the offset used in the meta-analysis models

| c | BA_vs_BACI | BA_CI | BA_p | CI_vs_BACI | CI_CI | CI_p | BAvsCI_p | BACI_over_BA | BACI_over_CI | var_ratio | logLik |
| --- | --- | --- | --- | --- | --- | --- | --- | --- | --- | --- | --- |
| 0.01 | -0.382 | [-0.58, -0.19] | 0.00012 | -0.288 | [-0.42, -0.15] | 2.3e-05 | 0.29 | 1.47 | 1.33 | 1002512 | -2103.3 |
| 0.1 | -0.396 | [-0.58, -0.21] | 2.4e-05 | -0.32 | [-0.45, -0.19] | 8.9e-07 | 0.36 | 1.49 | 1.38 | 10346 | -1768.1 |
| 0.25 | -0.399 | [-0.57, -0.23] | 2.7e-06 | -0.329 | [-0.45, -0.21] | 5.1e-08 | 0.34 | 1.49 | 1.39 | 1946 | -1511.1 |
| 0.5 | -0.362 | [-0.5, -0.22] | 4E-07 | -0.282 | [-0.38, -0.18] | 6E-08 | 0.19 | 1.44 | 1.33 | 537 | -1227.2 |
| 1 | -0.267 | [-0.37, -0.16] | 3.8e-07 | -0.184 | [-0.26, -0.11] | 3E-06 | 0.062 | 1.31 | 1.2 | 186 | -837.7 |


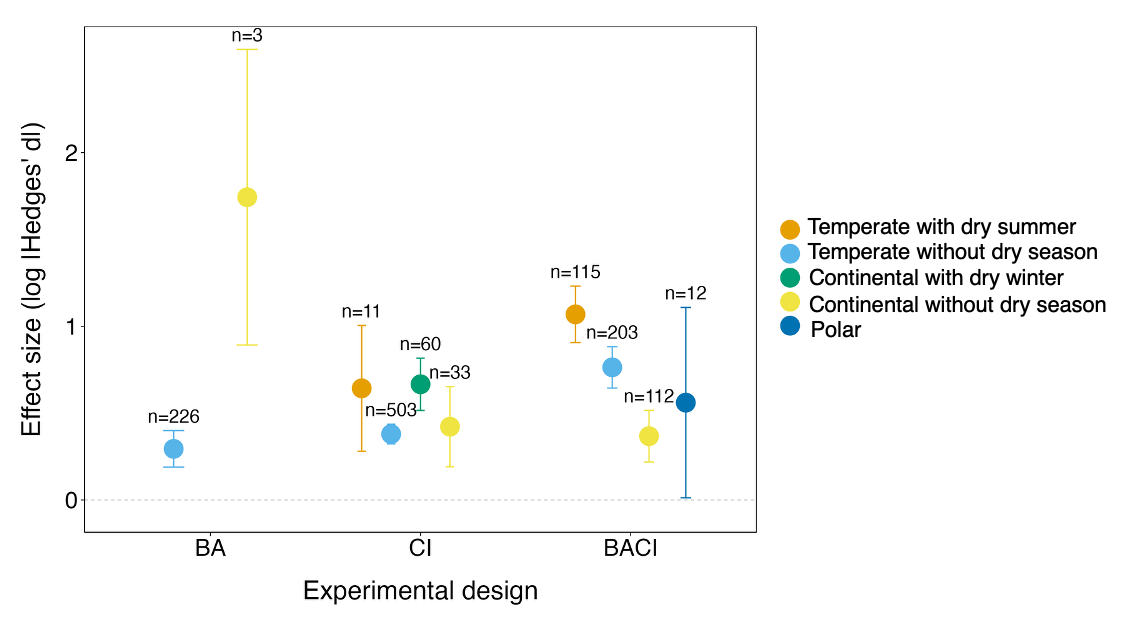


**Figure S3. Relationship between climate areas of studies and measured low-flow effects.** Plot showing Effect size estimates (log |Hedges’*d*|) and their corresponding 95% confidence interval by study design and climate area. The number of Effect sizes for each category is shown above each confidence interval.

For BA designs, impact of low-flow was significantly weaker in temperate region without dry season than in continental region without dry season (*p*=0.001). However, the number of effects sizes in both categories are really unbalanced, which makes this result rather weak. For CI design, impact of low-flow was not significantly different between different climates (*p*> 0.05). For CI design there was a tendency for climate with dry season (temperate and continental) to be more impacted by low-flow, even if these results were not significant. Finally, for BACI design, in continental climate without dry season effect of low-flow were significantly lower than in temperate climate, both with dry summer and without dry season (*p*<0.0001).


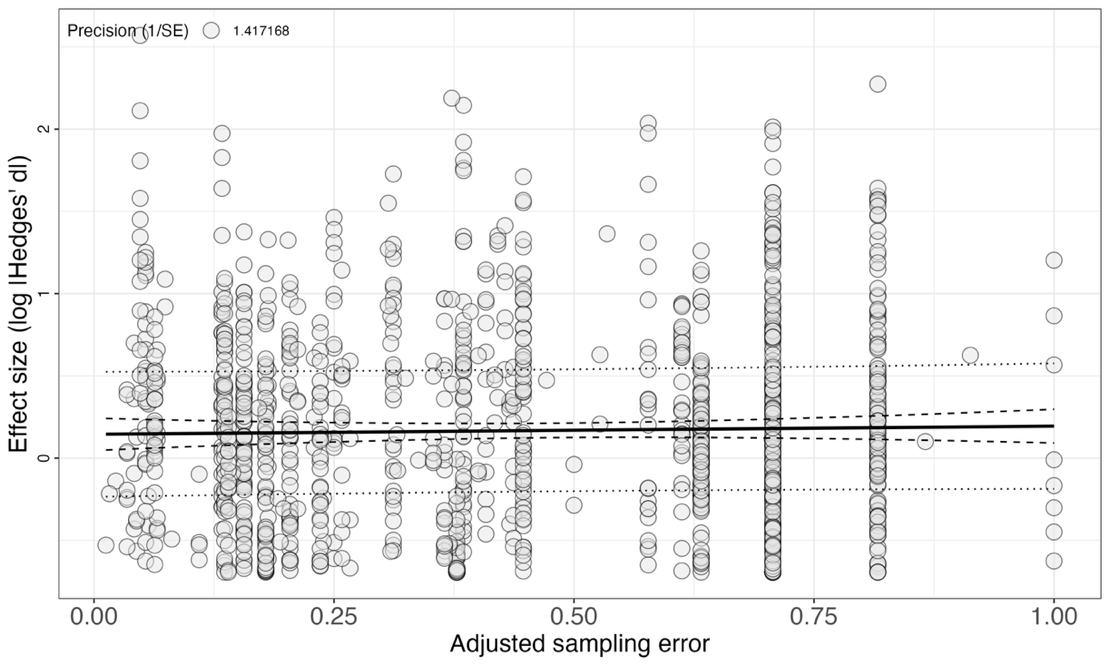


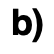


| Moderator | Design | Coefficient | 95% Confidence Interval | p-value |
| --- | --- | --- | --- | --- |
| - | All | 0.064 | [-0.108; 0.236] | 0.464 |
| Design | All | 0.761 | [0.537; 0.984] | **<0.0001** |
| Duration | All | 0.030 | [-0.145; 0.205] | 0.736 |
| Discharge | All | -0.065 | [-0.654; 0.513] | 0.513 |
| Velocity | All | 0.340 | [0.022; 0.658] | **0.036** |
| Response | All | 0.043 | [-0.136; 0.222] | 0.636 |
|  | BA | 0.061 | [-1.578; 1.697] | 0.942 |
|  | CI | 0.909 | [0.651; 1.167] | ***<0.0001*** |
|  | BACI | 1.008 | [0.243; 1.773] | **0.010** |
| Ecological component | All | 0.018 | [-0.164; 0.199] | 0.850 |
|  | BA | 0.776 | [-1.003; 2.554] | 0.393 |
|  | CI | 0.706 | [0.426; 0.941] | **<0.0001** |
|  | BACI | 0.943 | [0.199; 1.687] | **0.013** |
| Biological organization | All | 0.017 | [-0.164; 0.198] | 0.851 |
|  | BA | -0.739 | [-1.911; 0.432] | 0.216 |
|  | CI | 0.883 | [0.570; 1.197] | **<0.0001** |
|  | BACI | 0.780 | [-0.001; 1.562] | **0.050** |
| Climate | All | 0.320 | [0.141; 0.499] | **0.001** |
|  | BA | 0.270 | [-1.565; 2.104] | 0.773 |
|  | CI | 0.835 | [0.592; 0.835] | **<0.0001** |
|  | BACI | 1.501 | [0.717; 2.294] | **0.001** |

**Figure S4**. (a) Bubble plot showing the relationship between the effect sizes, calculated as the log |Hedges’d| and the adjusted sampling error, measured as √(1/𝑛̃), where 𝑛̃ represents the effective sample size of each effect size. (b) Table listing the values of the small studies effect (√(1/𝑛̃)) for all the models used. Significant p-values are written in bold.

**
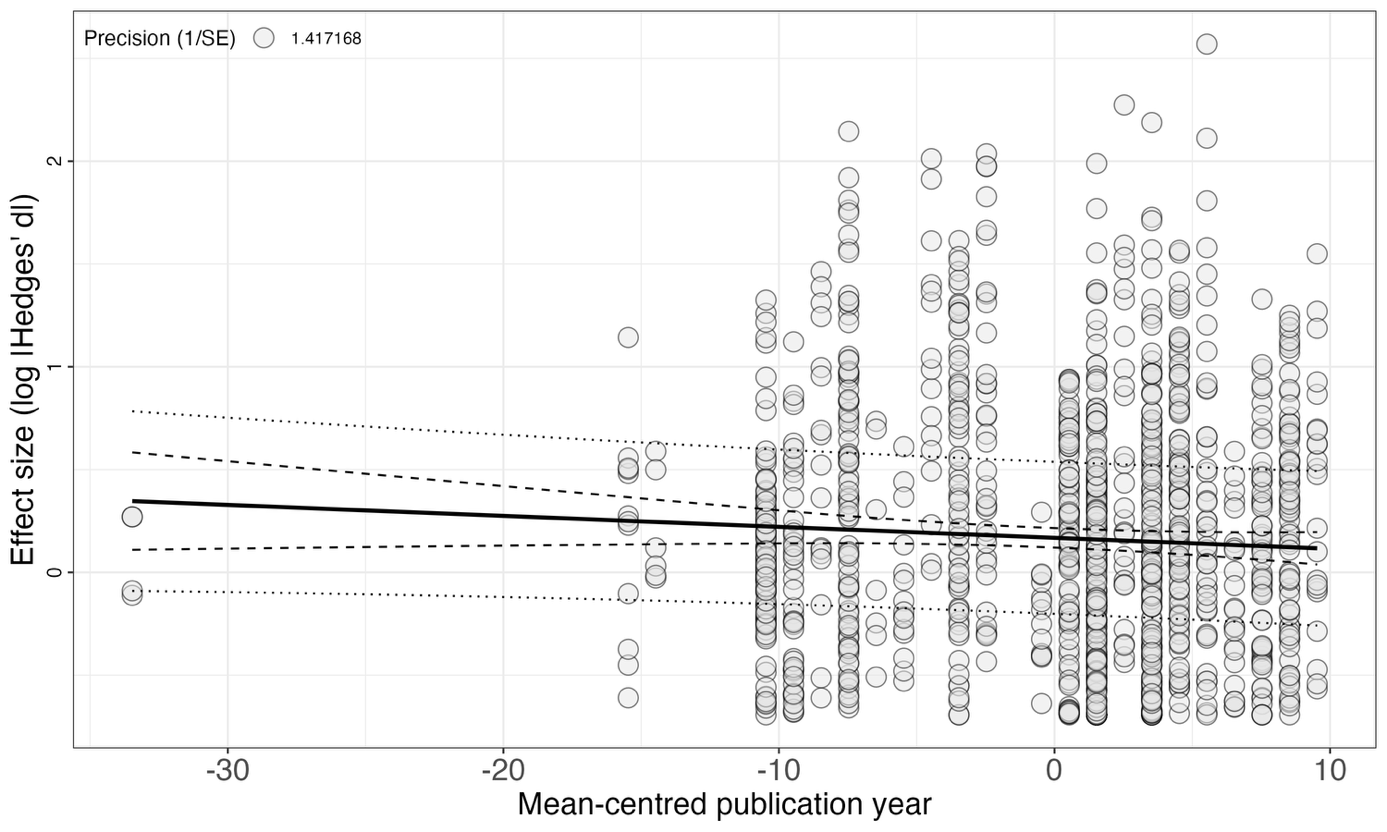
**

**Figure S5**. Bubble plot showing the relationship between the effect sizes, calculated as the log |Hedges’*d*| and the mean-centred publication year.

**Table S3.** Table showing the values of total, between and within studies heterogeneity (%) for each meta-analysis model.

| Moderator | Total heterogeneity | Between studies heterogeneity | Within studies heterogeneity |
| --- | --- | --- | --- |
| Design | 63.145 | 44.087 | 19.058 |
| Drought duration | 64.393 | 46.315 | 18.078 |
| Discharge reduction | 59.355 | 40.209 | 19.146 |
| Velocity reduction | 69.981 | 64.118 | 5.862 |
| Response type | 66.375 | 46.916 | 19.459 |
| Ecosystem endpoint | 63.640 | 43.724 | 19.916 |
| Level of biological organization | 64.703 | 44.191 | 20.512 |
| Climate | 62.251 | 44.738 | 19.513 |


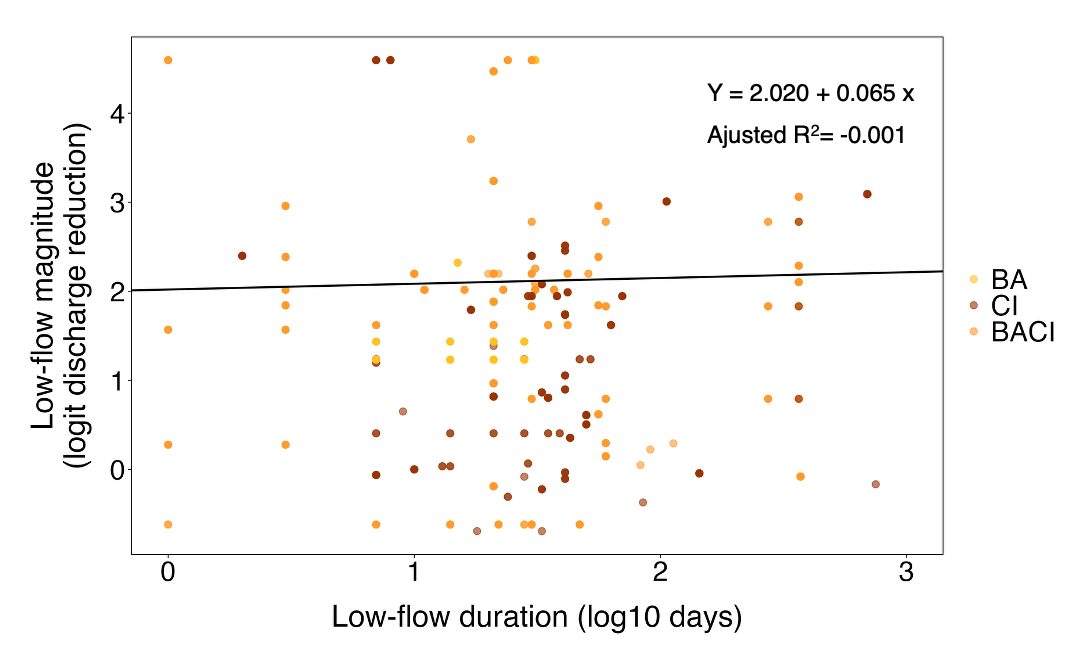


**Figure S6**. Scatter plot showing relationships between the duration of the low-flow period, measured as log10(day) and magnitude of discharge reduction, measured as logit (percentage of discharge reduction).


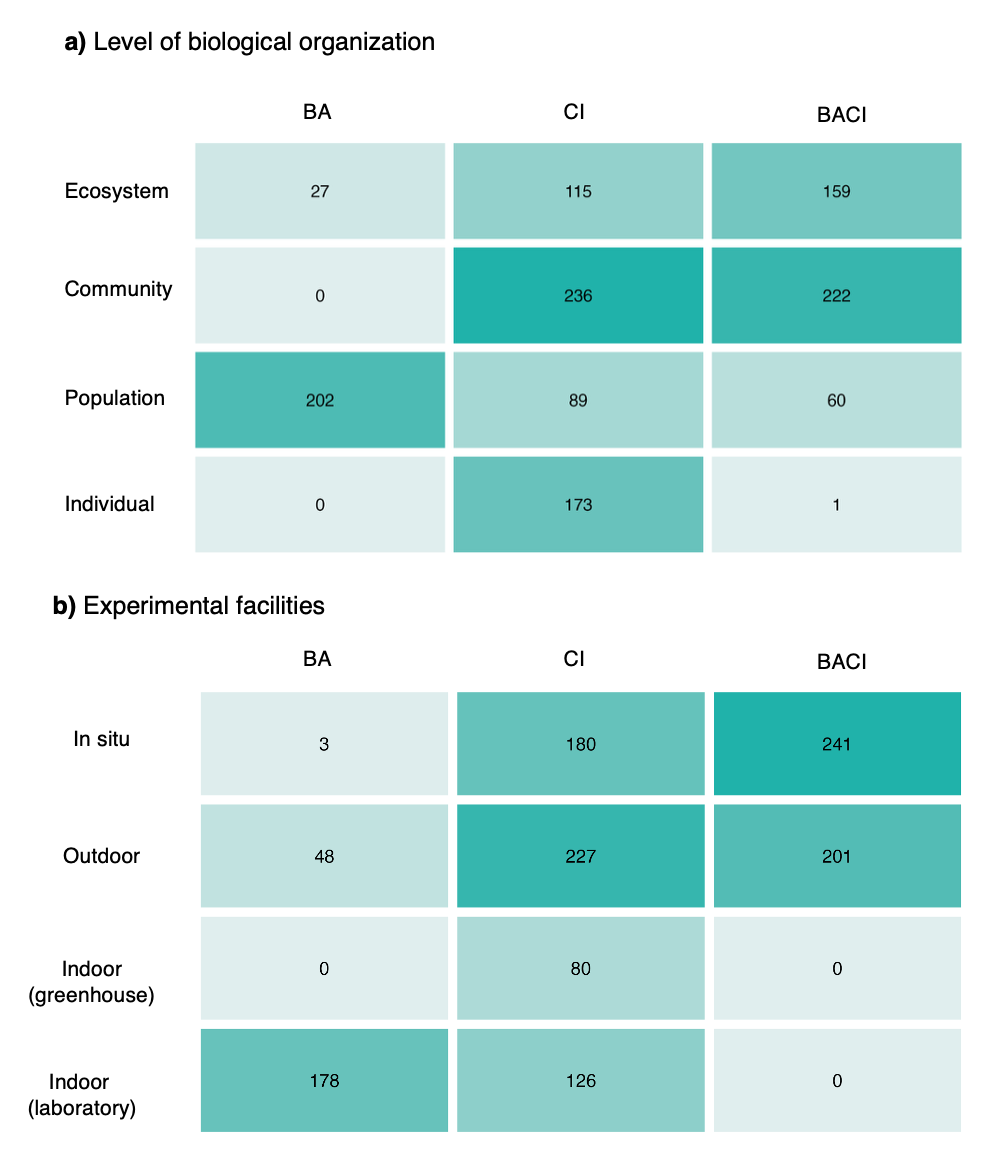


**Figure S7.** Heatmap showing the number of effect sizes in different categories of level of (a) biological organization and (b) experimental facilities for BA, CI and BACI studies.

These plots show that the data are unevenly distributed among the designs, which could indeed influence the differences in effect magnitudes observed between them. Nonetheless, a similar heterogeneity is apparent between BA and CI designs, for which we still do not detect any significant difference in effect size.
